# Cuproptosis/ferroptosis-related gene signature is correlated with immune infiltration and predict the prognosis for patients with breast cancer

**DOI:** 10.3389/fphar.2023.1192434

**Published:** 2023-07-13

**Authors:** Jixian Li, Wentao Zhang, Xiaoqing Ma, Yanjun Wei, Fengge Zhou, Jianan Li, Chenggui Zhang, Zhe Yang

**Affiliations:** ^1^ Shandong Provincial Hospital, Shandong University, Jinan, Shandong, China; ^2^ Shandong Provincial Hospital, Shandong First Medical University, Jinan, Shandong, China; ^3^ Tumor Research and Therapy Center, Shandong Provincial Hospital Affiliated to Shandong First Medical University, Jinan, Shandong, China; ^4^ Radiotherapy and Minimally Invasive Group I, The Second Affiliated Hospital of Shandong First Medical University, Taian, Shandong, China; ^5^ Department of Radiation Oncology, Weifang People’s Hospital, Weifang, China; ^6^ Department of Orthopedics, Shandong Provincial Hospital Affiliated to Shandong First Medical University, Jinan, Shandong, China

**Keywords:** cuproptosis, ferroptosis, BRCA, prognosis, immune

## Abstract

**Background:** Breast invasive carcinoma (BRCA) is a malignant tumor with high morbidity and mortality, and the prognosis is still unsatisfactory. Both ferroptosis and cuproptosis are apoptosis-independent cell deaths caused by the imbalance of corresponding metal components in cells and can affect the proliferation rate of cancer cells. The aim in this study was to develop a prognostic model of cuproptosis/ferroptosis-related genes (CFRGs) to predict survival in BRCA patients.

**Methods:** Transcriptomic and clinical data for breast cancer patients were obtained from The Cancer Genome Atlas (TCGA) and Gene Expression Omnibus (GEO) databases. Cuproptosis and ferroptosis scores were determined for the BRCA samples from the TCGA cohort using Gene Set Variation Analysis (GSVA), followed by weighted gene coexpression network analysis (WGCNA) to screen out the CFRGs. The intersection of the differentially expressed genes grouped by high and low was determined using X-tile. Univariate Cox regression and least absolute shrinkage and selection operator (LASSO) were used in the TGCA cohort to identify the CFRG-related signature. In addition, the relationship between risk scores and immune infiltration levels was investigated using various algorithms, and model genes were analyzed in terms of single-cell sequencing. Finally, the expression of the signature genes was validated with quantitative real-time PCR (qRT‒PCR) and immunohistochemistry (IHC).

**Results:** A total of 5 CFRGs (ANKRD52, HOXC10, KNOP1, SGPP1, TRIM45) were identified and were used to construct proportional hazards regression models. The high-risk groups in the training and validation sets had significantly worse survival rates. Tumor mutational burden (TMB) was positively correlated with the risk score. Conversely, Tumor Immune Dysfunction and Exclusion (TIDE) and tumor purity were inversely associated with risk scores. In addition, the infiltration degree of antitumor immune cells and the expression of immune checkpoints were lower in the high-risk group. In addition, risk scores and mTOR, Hif-1, ErbB, MAPK, PI3K/AKT, TGF-β and other pathway signals were correlated with progression.

**Conclusion:** We can accurately predict the survival of patients through the constructed CFRG-related prognostic model. In addition, we can also predict patient immunotherapy and immune cell infiltration.

## Introduction

Breast cancer is the most common cancer in women worldwide (Dumas et al., 2020) and seriously endangers women’s lives and health. There were approximately 2.3 million new cases and 685,000 deaths in 2020 (Lei et al., 2021). Breast cancer is a significantly heterogeneous cancer (Yeo and Guan, 2017), and with the continuous development of treatments in the form of surgery, chemotherapy, radiotherapy, targeted therapy, endocrine therapy, etc. (Singh et al., 2021), the survival of breast invasive carcinoma (BRCA) patients has improved. However, some patients still face the risk of recurrence and death. Currently known clinical, pathological and molecular features cannot accurately predict the prognosis of patients; thus, we still urgently need new prognostic markers to evaluate the prognosis of patients and to guide treatment.

Cuproptosis (Tang et al., 2022) and ferroptosis (Dixon et al., 2012) are novel cell death modes that do not depend on apoptotic pathways. The main cause of ferroptosis is the continuous generation of lipid reactive oxygen species (ROS) caused by excessive intracellular iron (Wang et al., 2022). Once ROS levels exceeds the ROS ability to resist oxygen, the ROS will produce oxidative stress, which will damage mitochondria, the endoplasmic reticulum and nucleic acids in cells and finally lead to cell death. Studies have shown that abnormal regulation of ferroptosis is closely related to the occurrence of various human diseases, including ischemic organ damage (Yan et al., 2020), neurodegeneration (Jakaria et al., 2021) and tumors (Mou et al., 2019). However, induction of ferroptosis can prevent malignant progression of tumors in patients, inhibit conventional therapy-resistant cells, and enhance the effectiveness of immunotherapy (Xu et al., 2021). Interestingly, Suppressor of fused homolog (SUFU) inhibits ferroptosis sensitivity in breast cancer cells through the Hippo/YAP pathway (Fang et al., 2022). Cuprotosis is the latest form of programmed cell death to be discovered. Maintaining an adequate amount of copper plays an important role in maintaining function and homeostasis in all living organisms. Excess copper will interact with fatty acylated components in the tricarboxylic acid (TCA) cycle, resulting in abnormal aggregation of fatty acylated proteins and loss of Fe-S cluster proteins (Tsvetkov et al., 2022), at which point proteotoxic oxidative stress is activated, causing cells to undergo “copper toxicity,” which in turn leads to cell death (Li S-R. et al., 2022). In addition, this inhibition of the TCA cycle can affect ferroptosis. Thus, through the TCA cycle, both cuproptosis and ferroptosis can be affected (Gao et al., 2019).

In this study, we innovatively linked cuproptosis and ferroptosis. The CFRGs that can predict OS in BRCA patients were screened out by means of bioinformatics, and a proportional hazards regression model was constructed. Based on this model, we can effectively predict the prognosis of patients and help them personalize their treatments.

## Material and methods

### Data resources

The necessary data were acquired from The Cancer Genome Atlas (TCGA) (https://portal.gdc.cancer.gov/), comprising of RNA-sequencing (RNA-seq) data, clinical information, and simple nucleotide variation data from a total of 1182 BRCA patients. Fragments per kilobase million (FPKM) was selected for mRNA expression profiling. We also acquired RNA-seq data and clinical information from the National Center for Biotechnology Information (NCBI) Gene Expression Omnibus (GEO) database (https://www.ncbi.nlm.nih.gov/geo/, ID: GSE20685, GSE42568, GSE58812). Specifically, GSE20685 contained RNA-seq and clinical data of 327 breast cancer samples, GSE42568 contained RNA-seq and clinical data of 104 breast cancer samples and 17 normal breast samples, and GSE58812 contained RNA-seq and clinical data of 107 breast cancer samples. To ensure uniformity, the mRNA expression data in the TCGA and GEO databases were transformed into transcripts per million (TPM) data by log2 transformation. We downloaded a total of 65 ferroptosis-related genes from the Molecular Signatures Database (MSigDB, http://www.gsea-msigdb.org/gsea/msigdb/) and an additional 19 cuproptosis-related genes from the literature (Tsvetkov et al., 2022) (Ren et al., 2021) (Tao et al., 2021) (Selvaraj et al., 2005).

### CFRG identification based on GSVA and WGCNA

GSVA is an algorithm used to analyze gene expression data and functional annotation. It can display the differences in samples on different gene sets by calculating gene set scores. The algorithm does not require standardization and is very fast, so it is widely used in biomedical research. A GSVA of the GSE42568 samples was performed on ferroptosis and cuproptosis genomes to obtain ferroptosis and cuproptosis scores, respectively. The hub genes were screened by weighted gene coexpression network analysis (WGCNA) based on their scores. Scale-free and average connectivity analyses were performed on modules with different power values using the PickSoftThreshold function to set the soft threshold power to 5. Then, the corresponding dissimilarity matrix (1-TOM) and topological overlap matrix (TOM) were obtained. Pearson correlation analysis was performed on coexpression modules with cuproptosis and ferroptosis scores after TOM.

X-tile software is a software designed by Yale University to calculate the optimal cutoff value for survival curves. We calculated the optimal cutoff values for the cuproptosis score and ferroptosis score in the TCGA cohort with this software and divided the scores into high- and low-score groups, respectively. Subgroups with both high and low scores for both cuproptosis and ferroptosis were selected. According to specific criteria (|log2FC| ≥ 1 and FDR<0.05), the “DeSeq2” R package was used to screen out the high and low subgroup differentially expressed genes (DEGs), and then the DEGs were cross-referenced with the genes screened by WGCNA, and the final genes were identified as CFRGs.

### Establishment and validation of the CFRGs prognostic model

After screening out CFRGs related to prognosis, we used five machine learning methods—decision tree, random forest, least absolute shrinkage and selection operator (LASSO), extreme gradient boosting (XGBoost) and gradient boosting decision tree (GBDT)—to evaluate the survival weights of the CFRGs. GSE42568 was used as the training set, and the first 30 genes were selected to construct the LASSO Cox model. With the median risk score as the critical value, the model was divided into a high-risk group and a low-risk group. The overall survival (OS) of the high and low risk groups was analyzed using the KM curve. Subsequently, Kaplan‒Meier (KM) analysis was performed on the high- and low-risk groups using the “survival” and “survminer” R packages, and 1-, 3-, and 5-year receiver operating characteristic (ROC) analyses were performed with the “timeROC” R package to evaluate the sensitivity of the model. Finally, the TCGA and GEO cohorts (GSE20685, GSE58812) were used as the validation set, and the obtained prognoses were selected for validation. In addition, we constructed a nomogram by combining the risk score and clinical data.

### Analysis of signature and immune-related markers

We used seven algorithms from the Tumor Immune Estimation Resource (TIMER) database, including TIMER, CIBERSORT, CIBERSORT-ASB, QUANTISEQ, MCPCOUNTER, XCELL, and EPIC, to analyze signatures and immunity. In addition, we evaluated the differences in immune cell infiltration and immune function between high-risk and low-risk subgroups using the single-sample gene set enrichment analysis (ssGSEA) algorithm. Furthermore, differences between high- and low-risk groups in immune therapy biomarkers were also analyzed through TIDE (http://tide.dfci.harvard.edu/)), TMB, and immune checkpoint.

### Characterization of OCFRGs (optimal cuproptosis/ferroptosis related genes) by single-cell RNA sequencing

Single-cell RNA-seq data for 12 samples were obtained from the GSE149655 dataset in the GEO database. The sequencing data were analyzed using the “Seurat” R package, and low-viability cells were removed. Using the CreateSeuratObject function, and based on specified criteria, low-quality cells were further removed (min.cells<3, min. features<50, percent. mt < 50). The filtered single-cell RNA sequencing (scRNA-seq) data were then normalized, and 1,500 genes with large coefficients of variation between cells were generated and subsequently subjected to principal component analysis (PCA) for dimensionality reduction of the scRNA-seq data. The top 4 PCs were selected for cell clustering analysis using the distributed stochastic neighbor embedding (t-SNE) algorithm. Through the DEGs of each cluster, the cell clusters were tool annotated. Finally, we analyzed the expression levels of OCFRGs in different clusters.

### Enrichment analysis of GSEA and GSVA

DEGs in high-risk and low-risk subgroups were screened and GSEA was performed using the GSEA software (https://www.gsea-msigdb.org/gsea/index.jsp). In addition, GSVA was used to perform GSVA on high-risk and low-risk subgroups using the “GSVA” R package. We collected some pathways closely related to the occurrence and development of tumors from the literature, including Hippo, Wnt, MAPK, PI3K/AKT, TGF-β, NF-kB, Notch, AMPK, JAK-STAT, PD-1/PD-L1, mTOR, Ras, TNF, HIF-1, ErbB, Nrf2, as well as functions and pathways related to cuproptosis and ferroptosis, including GLUTATHIONE PEROXIDASE ACTIVITY, P53 SIGNALING PATHWAY, GLUTATHIONE METABOLIC PROCESS, LIPID HOMEOSTASIS, PENTOSE PHOSPHATE PATHWAY, TRICARBOXYLIC ACID CYCLE, MITOCHONDRIAL TRICARBOXYLIC ACID CYCLE ENZYME COMPLEX, TRICARBOXYLIC ACID CYCLE ENZYME COMPLEX, REGULATION OF OXIDATIVE STRESS INDUCED CELL DEATH, RESPONSE TO OXIDATIVE STRESS, CELL MIGRATION INVOLVED IN SPROUTING ANGIOGENESIS, IRON METABOLISM. We used GSVA to calculate the enrichment score for each pathway and evaluate the correlation between risk score and sex enrichment score.

### Risk subtype analysis of drug sensitivity

In order to assess the sensitivity of high and low risk groups to drugs, we used the “pRRophetic” R package to analyze the RNA-seq data of BRCA based on the Genomics of Drug Sensitibity in Cancer (GDSC) database. The level of half maximal inhibitory concentration (IC50) reflects the patient’s sensitivity to drugs.

### Immunohistochemistry validation of the protein expression levels of OCFRGs

Five breast invasive ductal carcinoma tissue chips were purchased from Shanghai Outdo Biotech Company (Shanghai, China). Each tissue chip includes 45 cancer tissues and 45 paracancerous tissues. ANKRD52 (rabbit polyclonal, catalog number: GTX32443, Genetex), HOXC10 (rabbit polyclonal, catalog number: ab153904, Abcam), KNOP1 (rabbit polyclonal, catalog number: ab126512, Abcam), SGPP1 (rabbit polyclonal, catalog number: ab126512, Abcam): ab126512, Abcam) and TRIM45 (rabbit polyclonal, catalog number: TA505920, origene). The results of the immunohistochemical staining were scored. Semiquantitative scoring was performed according to the staining intensity and the percentage of positive cells: No staining, pale yellow (light yellow particles), medium (brown yellow particles), and heavy (dark brown particles) were scored as 0, 1, 2, and 3, respectively. According to the percentage of positively stained cells in the total number of cells, 0% was scored as 0, 5%–25% was scored as 1; 26%–50% was scored as 2; 51%–75% was scored as 3; and >75% was scored as 4 points. The final score was the sum of the staining intensity and the percentage of positive cells. The sum of the staining intensity and the percentage of positive cells was less than 6 for the low expression group and ≥6 for the high expression group. Five 400x high-power fields were randomly selected for each sepction, the staining intensity and percentage of positive cells were scored in each field, and the average value was calculated. The immunohistochemical staining results were microscopically adjudicated by two pathologists in an independent, double-blind manner.

### Quantitative real-time PCR

Normal breast epithelial MCF-10A cells and three human breast cancer cell lines, SK-BR-3, MDA-MB-231, and MCF-7, were obtained from the Central Laboratory of Shandong Provincial Hospital. Total RNA was extracted using TRIzol reagent (Invitrogen, United States). Complementary DNA (cDNA) was synthesized using the PrimeScript RT kit (Takara). Supplementary Table S2 shows the primer sequences for Quantitative real-time PCR analysis of OCFRGs.

### Western blotting

Cells were lysed in cold Radioimmunoprecipitation assay (RIPA) buffer. The same amount of protein was subjected to SDS-PAGE, and then transferred to PVDF (polyvinylidene fluoride) membrane. Block with nonfat dry milk containing TBST for 1 h. The primary antibody (Western blot and IHC universal primary antibody) was diluted according to the instructions and incubated overnight at 4°C. After washing with TBST, the secondary antibody was added and incubated for 1 h at room temperature. After washing the membrane, it was developed using enhanced chemiluminescence (ECL) chromogenic solution.

### Statistical analysis

The bioinformatics analysis, statistical analysis, and machine learning aspects of this study were all performed with R software (version 4.0.1). Statistical tests included the Pearson chi-square test and Wilcoxon rank-sum test. Kaplan-Meier analysis was used to assess OS in both groups of patients. *p* < 0.05 was considered to be statistically significant.

## Results

### Identification of cuproptosis/ferroptosis-related genes

**FIGURE 1:**
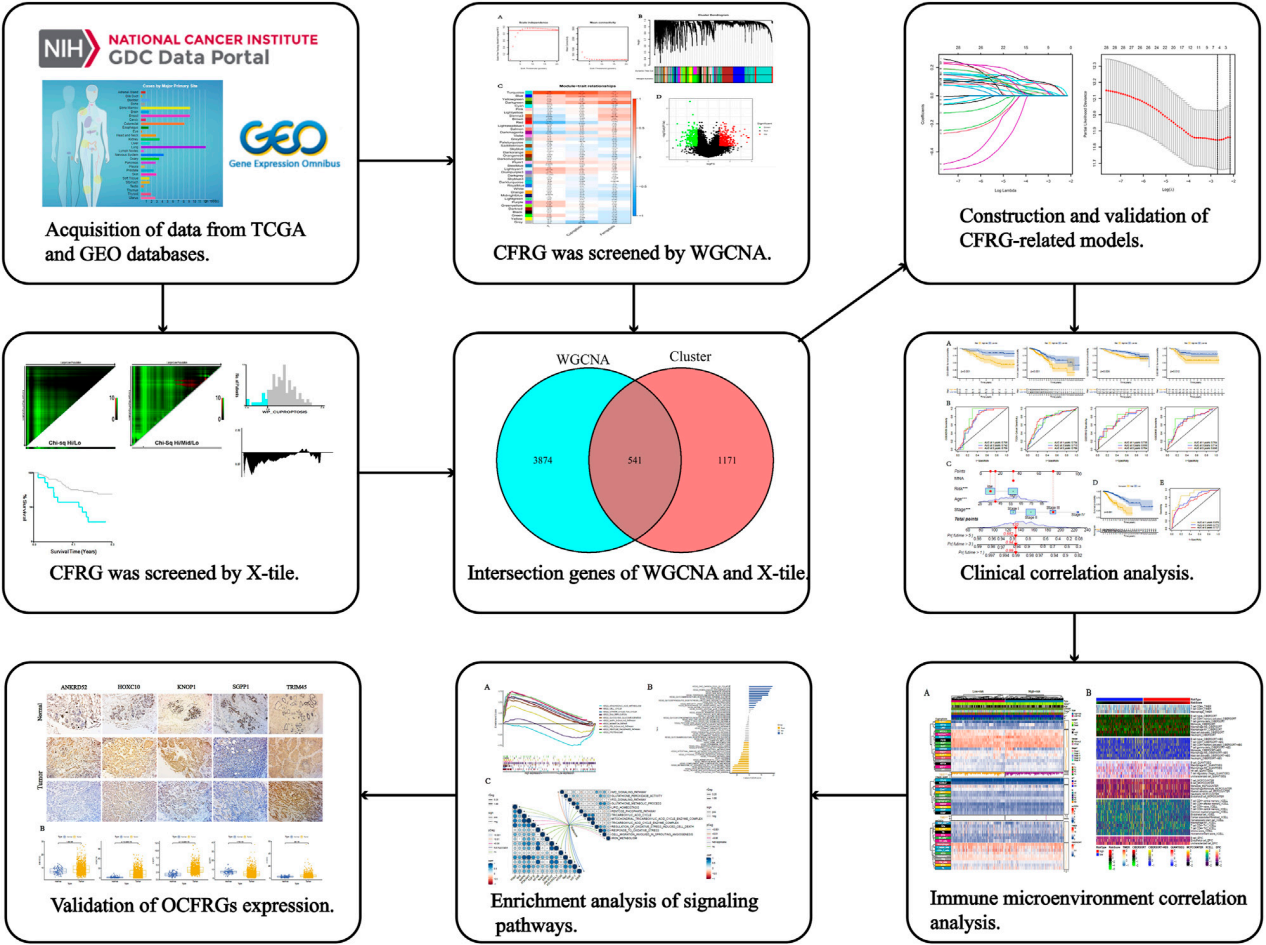
The workflow of the research. The figure shows the process of constructing and analyzing the DFRG-related signature.

The specific process of this study is shown in Figure 1, which includes the acquisition of data, the acquisition of cuproptosis and ferroptosis-related genes, the construction and validation of the signature, the analysis of clinical data, the analysis of immune cell infiltration levels, and the analysis of pathways and functions.

**FIGURE 2:**
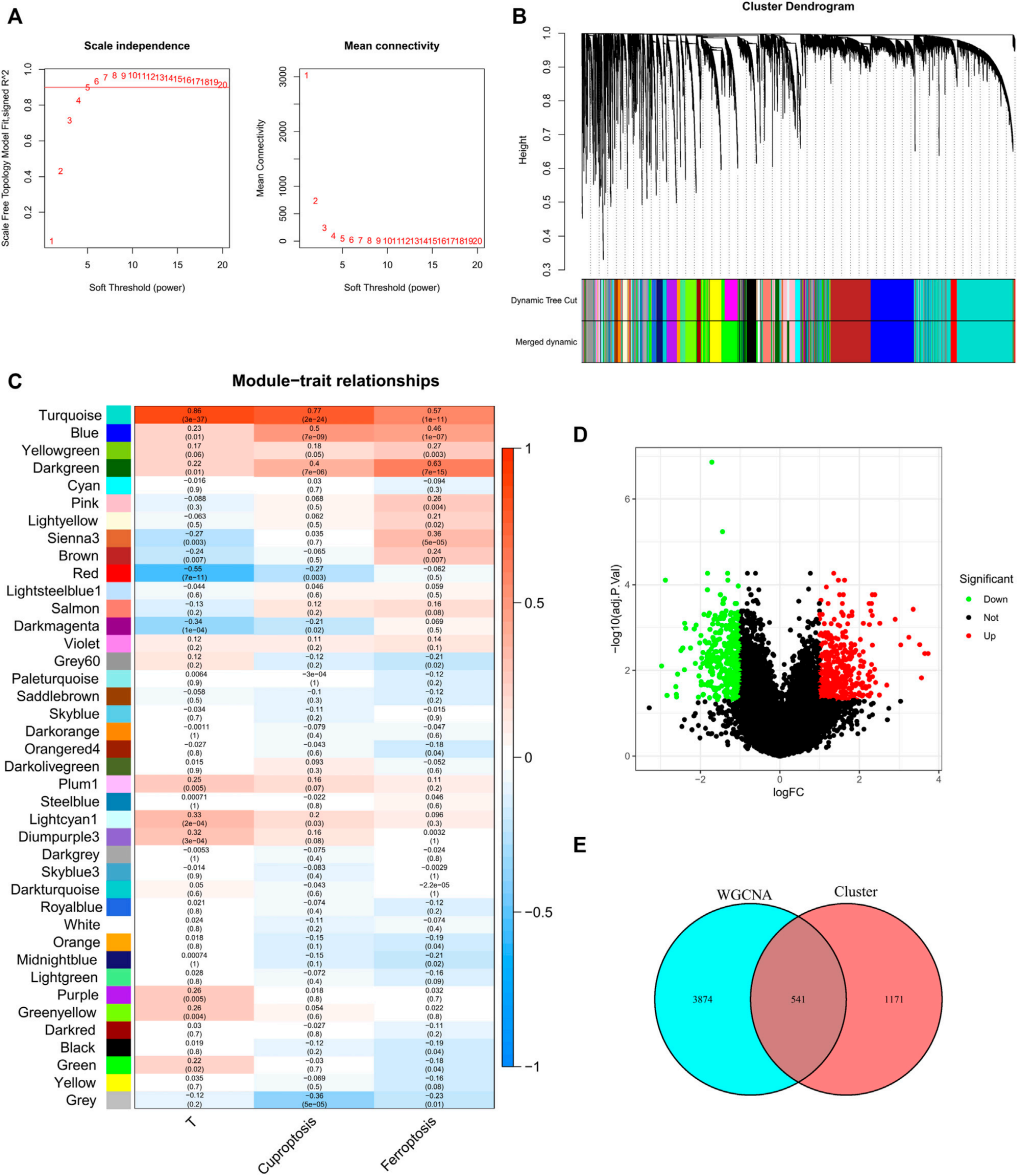
Discovery of prognostic CFRGs by WGCNA and Score grouping. **(A)** The distribution and trends of scale free topology model fit and mean connectivity along with soft threshold. **(B)** The clustering of gens among different modules by the dynamic tree cut and merged dynamic method. The gray modules represent unclassified genes. **(C)** Average correlation between multiple modules and tumor development, cuproptosis and ferroptosis. The color of the cell indicates the strength of the correlation, and the number in parentheses idicates the *p*-value for the correlation test. **(D)** According to specific criteria (*p* < 0.05, |Log2FC|≥1), 1712 DEGs wew screened for high and low score groups of cuproptosis and ferroptosis. **(E)** 541 CFRGs were obtained from WGCNA and high and low score groups.

We used the pickSoftThreshold function in the WGCNA R package to automatically select a soft threshold of 5 (Figure 2A). Multiple gene modules were divided by the dynamic cutting method, and the mergeCloseModules function was used to perform cluster analysis on each module into fewer modules and mark them with different colors (Figure 2B). To determine the correlation of coexpression modules with genetic differences between normal and tumor samples that may contribute to tumor development, cuprotosis score and ferroptosis score, we used Pearson correlation analysis, and the results showed that the module “turquoise” had the strongest correlation (R (Turmor) = 0.86, R (cuproptosis) = 0.77, R (ferroptosis) = 0.57) (Figure 2C).

X-tile software was used to determine the optimal cutoff values for the survival curves of the cuproptosis and ferroptosis scores in the TCGA BRCA cohort (Supplementary Figure S1). Patients with high cuproptosis and ferroptosis scores and patients with low cuproptosis and ferroptosis scores were divided into two groups, and DEGs were screened out with |log2FC| ≥ 1 and FDR<0.05 as criteria (Figure 2D). By analyzing the intersection of the genes screened by means of DEGs and WGCNA, a total of 541 CFRGs were identified (Figure 2E).

### Establishment and validation of the CFRG prognostic model

**FIGURE 3:**
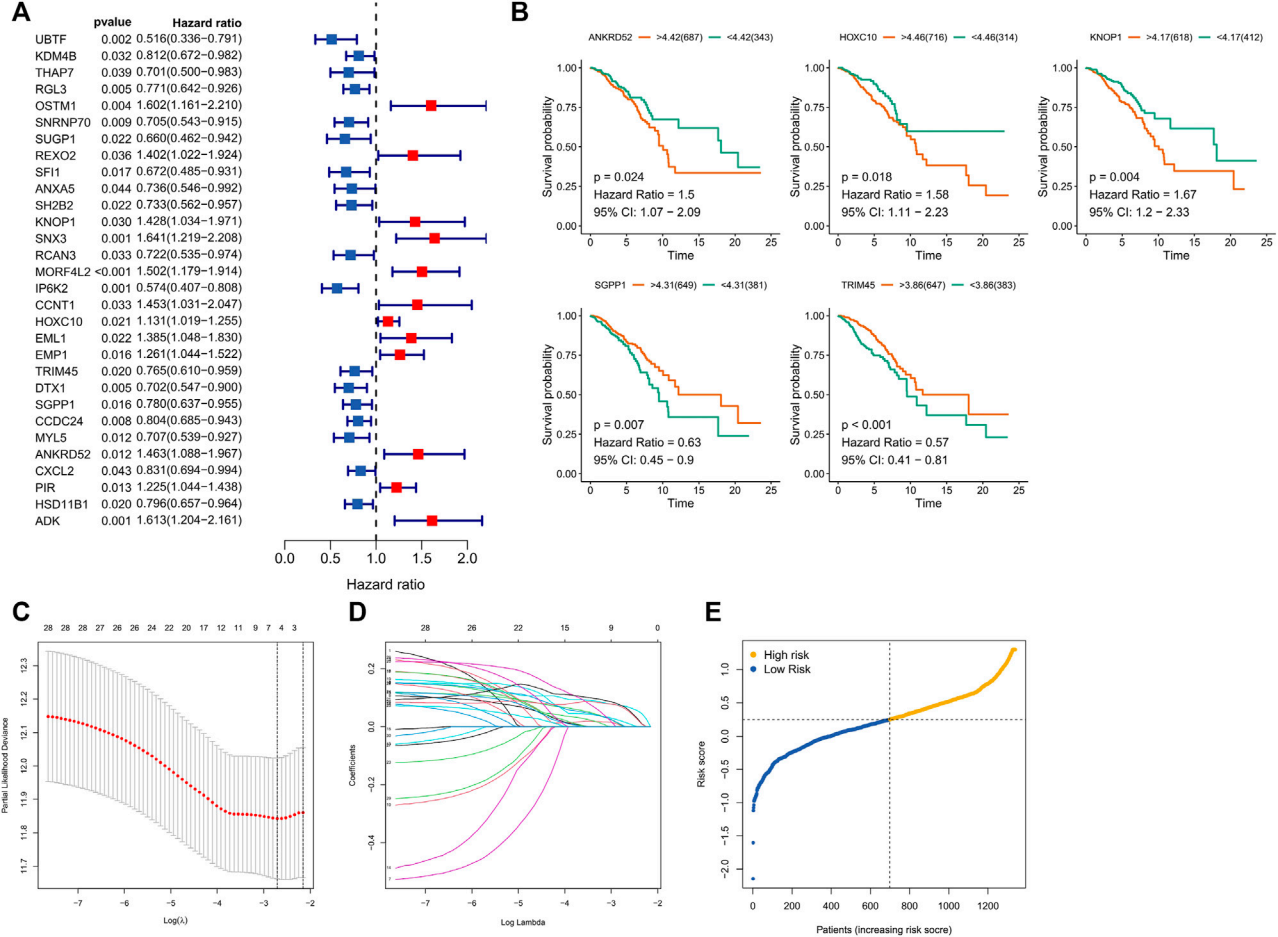
Screening and Models Construction of OCFRGs. **(A)** Forest plot of univariate Cox regression analysis (only showing the top 30 CFRGs related to survival). **(B)** Differences in survival between samples with high and low expression of OCFRGs (*p* < 0.05). **(C)** Selecting the best value of lambda through LASSO regression. **(D)** LASSO coefficient configuration of 5 OCFRGs. **(E)** Distribution of patients based on the median risk score in the training set.

Survival-related CFRGs were screened by using Cox regression analysis (Figure 3A). The survival-related weights of the CFRGs were evaluated using five machine learning algorithms, including decision tree, random forest, LASSO, XGBoost, and GBDT. The top 30 CFRGs with average scores (Supplementary Table S1) were selected to screen genes and to construct models by means of LASSO Cox regression analysis. According to the best λ value, 5 prognostic genes (Figure 3B) were selected and signature was constructed (Figure 3C,D). Calculation of the risk score was conducted as follows:

$$\begin{array}{l} Risk\;Score=\left(0.4024*ANKRD52\;\exp.\right)+\left(0.1025*HOXC10\exp.\right)\\ +\left(0.2146*KNOP1\exp.\right)+\left(-0.3069*SGPP1\;\exp.\right)+\left(-0.4003*TRIM45\;\exp.\right) \end{array}$$

**FIGURE 4:**
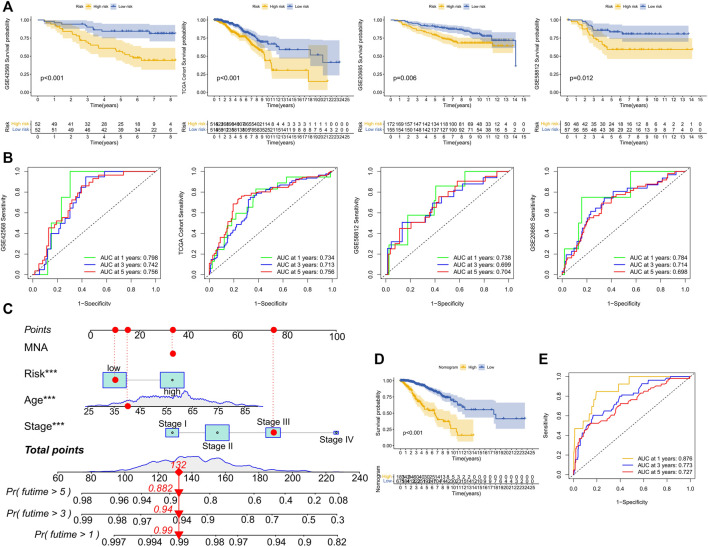
Performance evaluation of model prognosis and construction of nomogram. **(A)** KM curve shows the difference between high and low-risk groups in OS for GSE42568, TCGA cohort, GSE20685, and GSE58812. **(B)** ROC curves show the predictive efficiency of risk scores for GSE42568, TCGA cohort, GSE20685, and GSE58812 at 1, 3, and 5 years. **(C)** Column chart of overall survival prediction at 1 year, 3 years, and 5 years. The red line shows an example of how to predict prognosis. **(D)** The difference in survival between high and low-score groups in nomogram. **(E)** The predictive.

Based on the median training set risk score, BRCA patients were divided into high- and low-risk groups and further analyzed (Figure 3F). In the training and validation sets, the KM curve showed that the OS (Overall survival) in the high-risk group was lower than that in the low-risk group (Figure 4A), which was statistically significant (*p* < 0.05). The ROC curve of the training set showed that the AUCs at 1 year, 3 years, and 5 years were 0.76, 0.726, and 0.728, respectively. The AUC of the TCGA cohort was 0.734, 0.713, and 0.756, and that of GSE58812 was 0.784, 0.714, and 0.698. This indicates that our model has reliable predictive performance. We constructed a nomogram, in which Age (*p* < 0.001), Stage (*p* < 0.001), and risk score (*p* < 0.01) were all statistically significant (Figure 4C). In the nomogram, the OS of high-scoring patients was significantly lower than that of low-scoring patients (Figure 4D), which was statistically significant (*p* < 0.05). The column chart had good predictive performance with AUCs of 0.876, 0.773, and 0.727 at 1, 3, and 5 years, respectively.

### Clinical analysis of the signature

**FIGURE 5:**
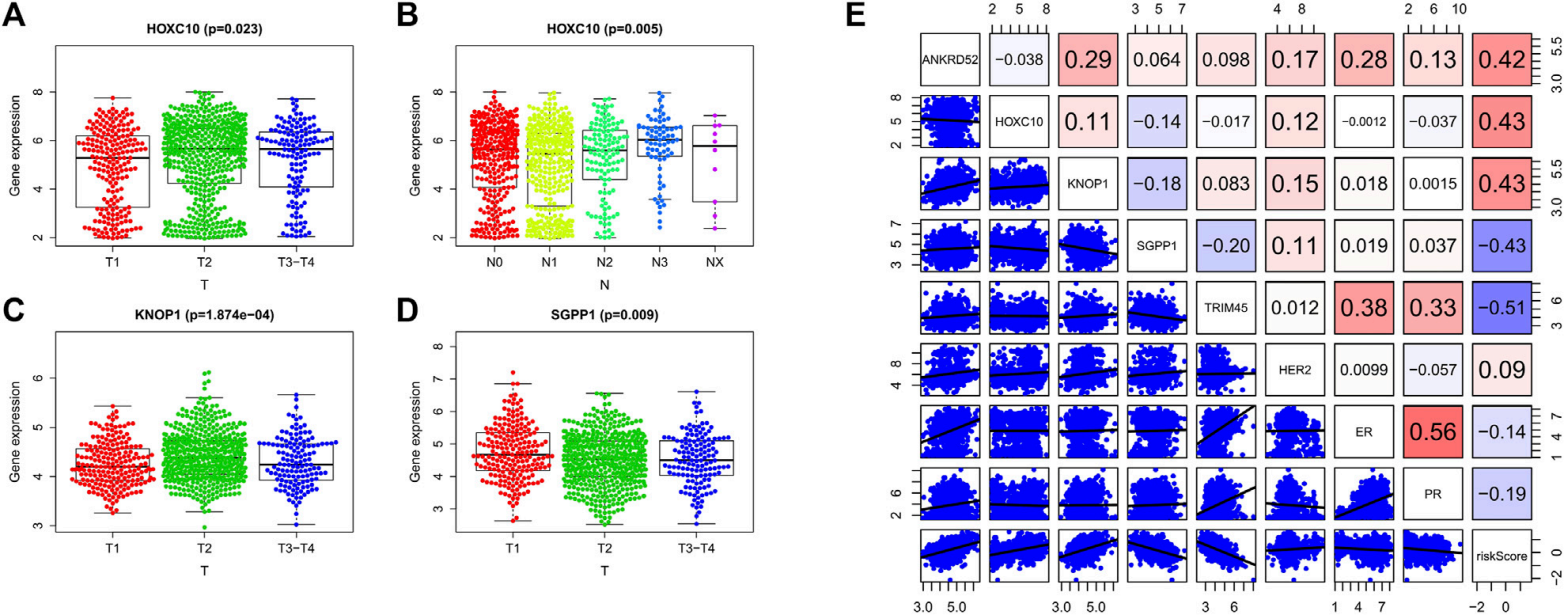
Clinical analysis of the model. **(A–D)** By Kruskal–Wallis test, the expression differences of OCFRGs (ANKRD52, HOXC10, KNOP1, SGPP1, TRIM45) in T stage and N stage were analyzed. **(E)** By calculating the Pearson correlation coefficient, the correlation between OCFRGs and the risk score and clinical indicators ER, PR, HER2 of breast cancer were analyzed.

**FIGURE 6:**
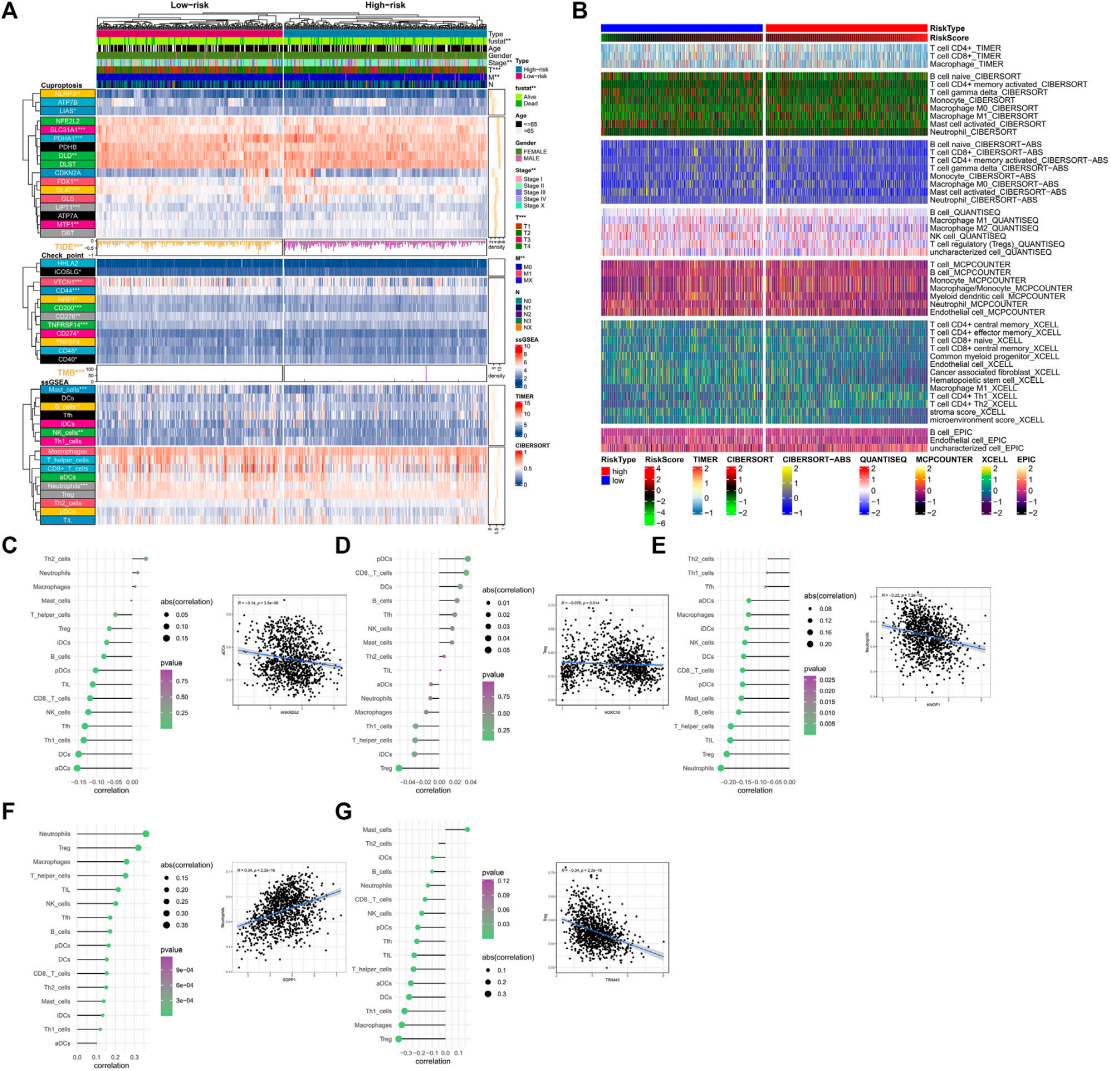
Analysis of immune cell infiltration related to the signature. **(A)** Differences in clinical data, CRGs, TMB, TIDE scores, check point and ssGSEA results between high and low-risk groups. TIDE Scores and TMB are presented in the form of a bar chart and density plot respectively. **(B)** The differences in the abundance of immune cell infiltration algorithms in TIMER2.0 database including TIMER, CIBERSORT, CIBERSORT-ABS, MCPCOUNTER, XCELLL and EPIC between the high-risk and low-risk groups (the heatmap shows the results with statistical differences). **(C–G)** The correlation between OCFRGs and immune infiltration level. The color represents the significance, with greener colors indicate higher significance. The size of the circles represents the correlation coefficient.

We analyzed the relationship between OCFRGs (ANKRD52, HOXC10, KNOP1, SGPP1, TRIM45) and clinical data. The results showed that the expression level of HOXC10 differed significantly in different T and N stages and was statistically significant. In addition, the higher the expression level of HOXC10, the later the T stage and N stage of the patient (see Figures 5A,B). The expression levels of KNOP1 and SGPP1 also differed significantly in different T stages (see Figures 5C,D) and were statistically significant. We also analyzed the correlation between the expression levels of OCFRGs, risk score, and the expression levels of ER, PR, and HER2 (see Figure 5E). The results showed that the expression level of ANKRD52 was positively correlated with ER (R = 0.28). TRIM45 was also positively correlated with ER (R = 0.38) and PR (R = 0.33). There were also differences in stage, T stage, and M stage between the high-risk and low-risk groups (Figure 6A).

### Analysis of risk subtypes and immune markers

We analyzed the relationship between several immune-related markers and the risk subtypes. The results showed that the expression of numerous immune checkpoints was different between high and low-risk groups (Figure 6A), especially CD274 (PDL1, Programmed Cell Death 1 Ligand 1). Additionally, TMB (tumor mutational burden) and TIDE (tumor immune dysfunction and exclusion) are considered important immune therapy markers. Our study found that TMB and TIDE were also different between high and low-risk groups. The results based on the ssGSEA algorithm showed that the expression levels of Mast cells, B cells, NK cells, and Neutrophils also differed between high and low-risk groups. Moreover, according to the results from TIMER database (Figure 6B), all immune-related markers had different expression levels between the high and low-risk groups. It is worth noting that all of the immune-related analyses had statistical significance (*p* < 0.05).

In terms of immune cell infiltration, the expression of OCFRGs was mainly correlated with the infiltration levels of neutrophils and regulatory T cells (Tregs). The expression of ANKRD52 was negatively correlated with the infiltration level of activated dendritic cells (aDCs) (R = −1.4, *p* < 0.001, Figure 6C), and the expression level of HOXC10 was negatively correlated with the infiltration level of Tregs (R = −0.076, *p* = 0.014, Figure 6D). The expression level of KNOP1 was negatively correlated with the infiltration level of neutrophils (R = −0.22, *p* < 0.001, Figure 6E), and the expression level of SGPP1 was positively correlated with the infiltration level of neutrophils (R = 0.34, *p* < 0.001, Figure 6F). The expression level of TRIM45 was positively correlated with the infiltration level of Tregs (R = −0.34, *p* < 0.001, Figure 6G).

### Overview of the scRNA-Seq data generated from BRCA

**FIGURE 7:**
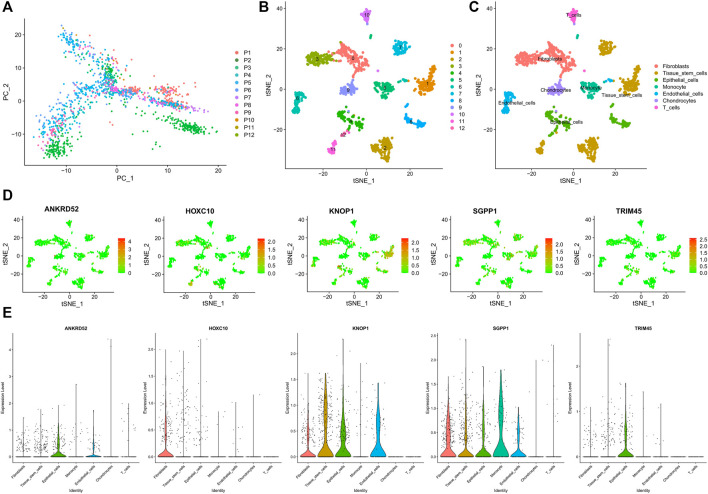
Verification of OCFRGs through sc-RNA seq. **(A)** PCA of scRNA-seq data for preliminary dimensionality reduction. **(B,C)** tSNE plots of cells generated from brest cancer tissue. The plots are colored by cell cluster, and the cells are clustered into seven sub-clusters. Each dot represents a breast cance cell. **(D)** The expression of signature genes in BRCA visualized in tSNE. **(E)** Violin plots depicting the expression of signature genes in clusters of BRCA. The y-axis shows the normalized read count. t-SNE:t-distributed stochastic neighbor embedding.

We obtained 14592 cells from 12 samples of GSE168410. A total of 1,418 cells were obtained by screening the total cells according to the intracellular gene features, the percentage of chromosomal genes, etc. Afterward, the top 10 genes with large coefficients of variation were labeled. Latitude reduction was performed using PCA (Figure 7A). The first 4 PCs were retained for further t-SNE, and 13 cell subsets were obtained (Figure 7B). The top 5 genes in each cluster were visualized with a heatmap (Supplementary Figure S3). We used tools to annotate the cell subsets, namely, fibroblasts, tissue stem cells, epithelial cells, monocytes, endothelial cells, chondrocytes, and T cells (Figure 7C). To investigate the expression of marker genes in different cells, we visualized them with t-SNE and violin plots (Figures 7D,E). OCFRGs were highly expressed in epithelial cells; HOXC10, KNOP1, and SGPP1 were highly expressed in fibroblasts; and only SGPP1 was highly expressed in monocytes.

### Analysis of function and signaling pathways

**FIGURE 8:**
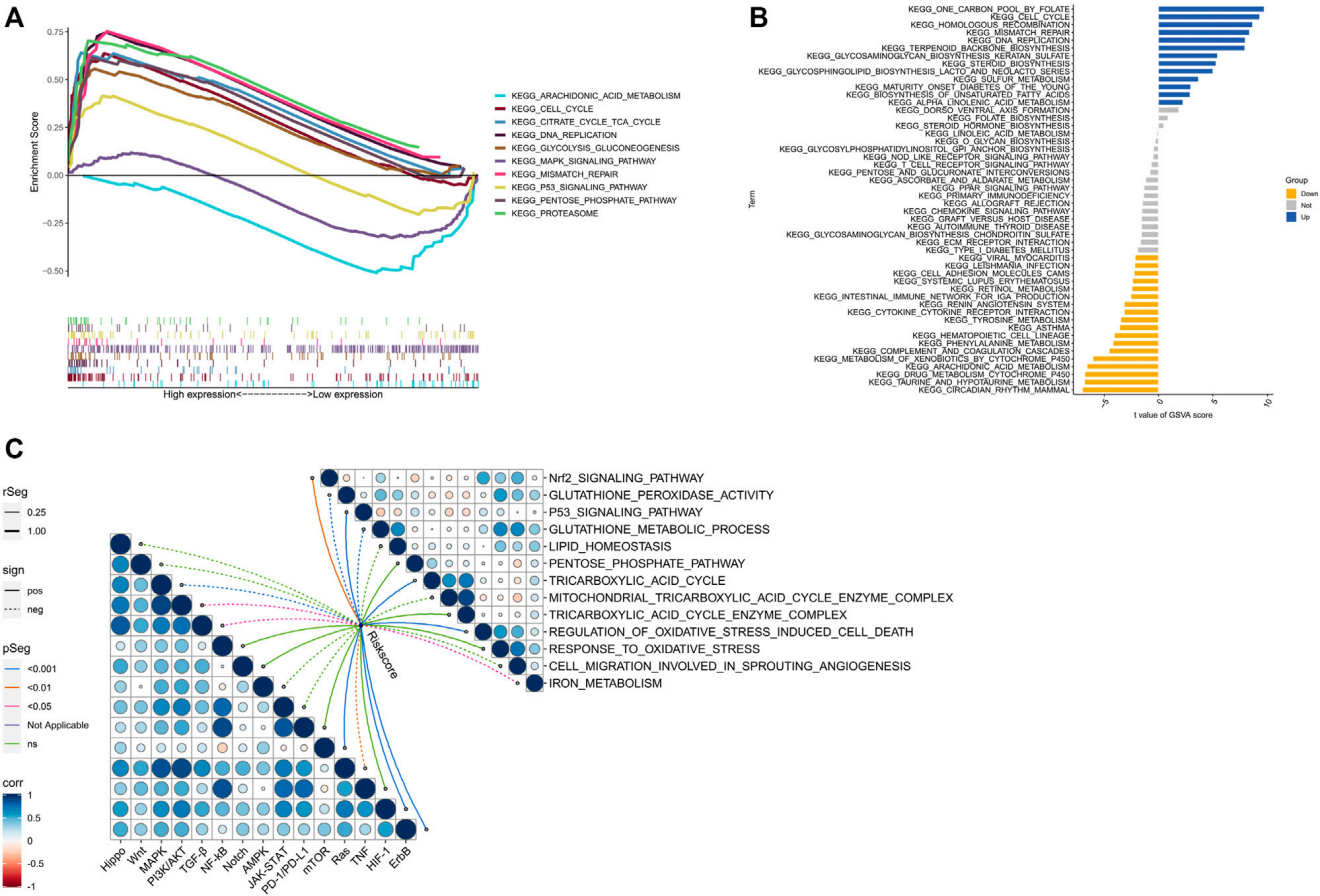
Biological functions. **(A)** Significantly enriched pathwways in the high-risk and low-risk groups. The extremum located on the left side idicates a psotive association between risk scores and pathway activity, and *vice versa*. **(B)** There is a significant difference in pathways between high-risk and low-risk groups. Blue bars represent a positive correlation between risk scores and pathway aactivity, while yellow bars indicate the opposite. **(C)** The correlation between riskscore and tumor important pathways, as well as cuproptosis/ferroptosis-related functions.

The biological functions of high- and low-risk groups were analyzed using GSEA software, which revealed that certain functions such as CELL CYCLE, CITRATE CYCLE/TCA CYCLE, DNA REPLICATION, GLYCOLYSIS/GLUCONEOGENESIS, MISMATCH REPAIR, PENTOSE PHOSPHATE PATHWAY, and PROTEASOME were more active in the high-risk group. On the other hand, the low-risk group exhibited significant enrichment in ARACHIDONIC ACID METABOLISM, MAPK SIGNALING PATHWAY, and P53 SIGNALING PATHWAY (Figure 8A). Additionally, GSVA analysis (Figure 8B) indicated that CELL CYCLE, HOMOLOGOUS RECOMBINATION, MISMATCH REPAIR, and DNA REPLICATION were more enriched in the high-risk group, whereas INTESTINAL IMMUNE NETWORK FOR IGA PRODUCTION, CYTOKINE CYTOKINE RECEPTOR INTERACTION, TYROSINE METABOLISM, and ARACHIDONIC ACID METABOLISM evidenced more enrichment in the low-risk group. Remarkably, several signaling pathways, including mTOR, HIF-1, and ErbB, were significantly activated in the high-risk group, whereas other signaling pathways such as MAPK, PI3K/AKT, TGF-β, and Ras were significantly inhibited (Figure 8C). Furthermore, the scores for cuproptosis/ferroptosis-related functional pathways, including Nrf2 SIGNALING PATHWAY, P53 SIGNALING PATHWAY, TRICARBOXYLIC ACID CYCLE, and REGULATION OF OXIDATIVE STRESS INDUCED CELL DEATH, were positively correlated with the risk score. In contrast, the scores for GLUTATHIONE PEROXIDASE ACTIVITY, GLUTATHIONE METABOLIC PROCESS, and IRON METABOLISM were negatively correlated with the risk score. All the aforementioned analyses exhibited statistical significance (*p* < 0.05).

### Drug sensitivity analysis between high and low risk groups

**FIGURE 9:**
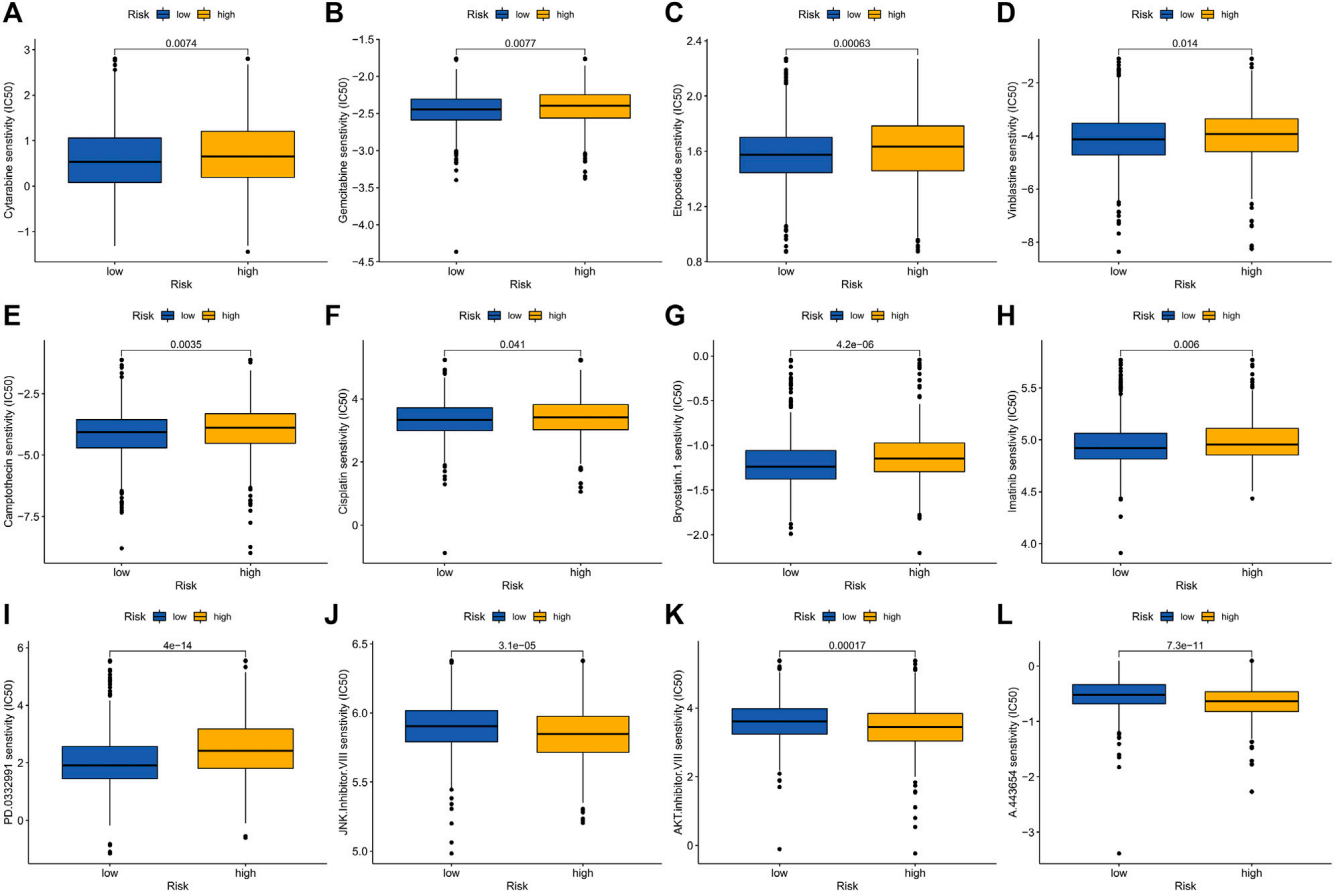
Drug sensitivity in patients in high and low risk groups. **(A)** Cytarabine, **(B)** Gemcitabine, **(C)** Epotoside, **(D)** Vinblastine, **(E)** Camptothecin, **(F)** Cisplatin, **(G)** Bryostatin.1, **(H)** Imatinib, **(I)** PD.0332991 (Palbociclib), **(J)** JNK. inhibitor.VIII, **(K)** AKT. Inhibitor.VII and **(L)** A.443654.

Elesclomol is a potent copper ionophore that promotes cuproptosis. Our analysis shows that the IC50 of various anticancer drugs including elesclomol is different in the high- and low-risk groups, and the IC50 values of high-risk groups are generally higher than those of low-risk groups (Figure 9A–L).

### Experimental verification of the expression of OCFRGs

**FIGURE 10:**
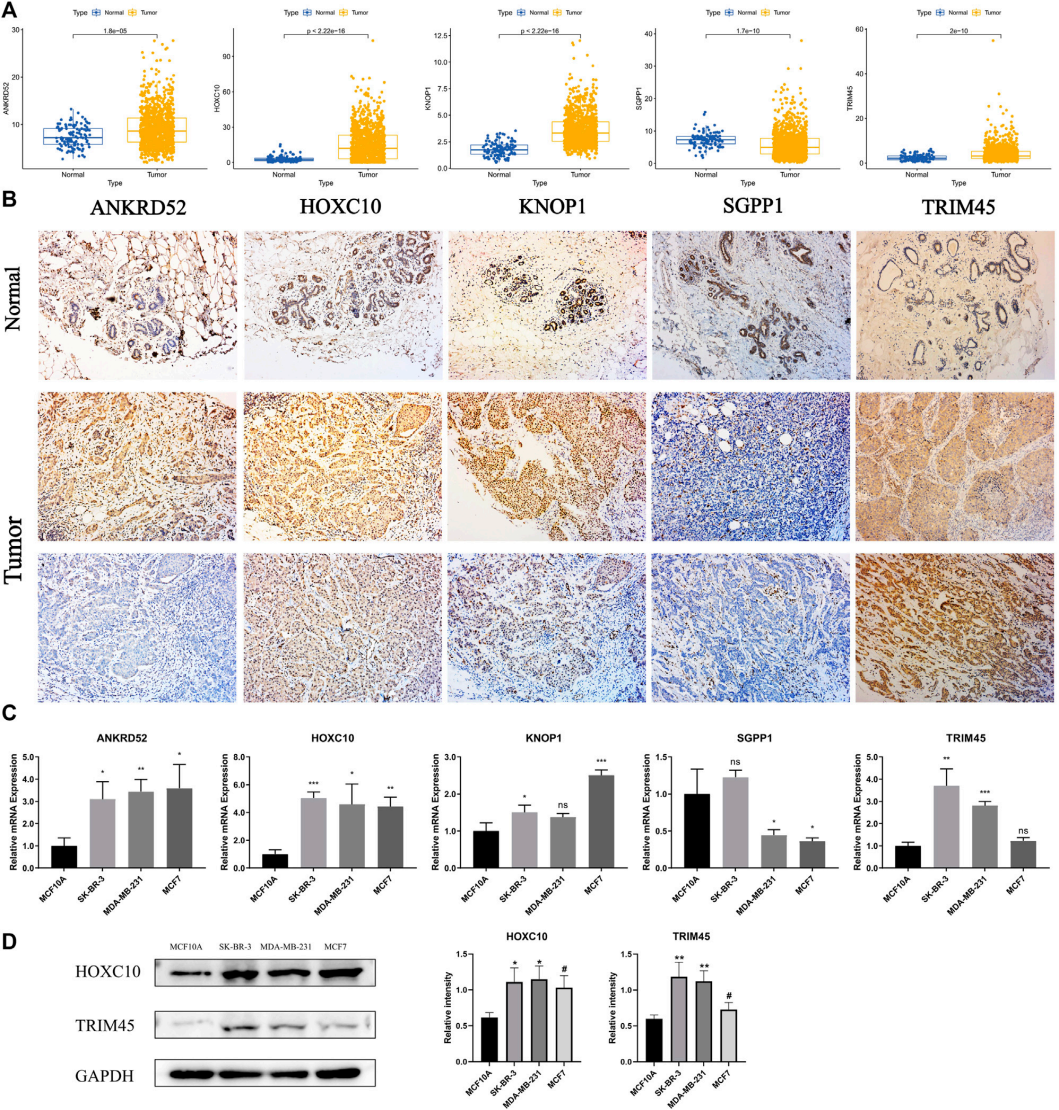
THe mRNA and Immunohistochemistry of OCFRGs expression. **(A)** Differences in mRNA levels of OCFRGs between normal and various tumor cells. **(B)** Immunohistochemical staining OCFRGs in BRCA and adjacent tissues. **(C)** Pcr verified the expression levels of OCFRGs in normal and tumor cells. **(D)** Western blotting verified the expression levels of OCFRGs in normal and tumor cells.

To explore the differences in the protein expression of OCFRGs in normal tissues and adjacent tissues, we detected the expression levels of ANKRD52, HOXC10, KNOP1, SGPP1, and TRIM45 in tissues by using immunohistochemical staining. The results of ANKRD52 level expressed that the HOXC10, KNOP1, and TRIM45 in the tumor tissue were higher in the paracancerous tissue, and the opposite was true for SGPP1 (Figure 10A). qRT-PCR results showed that ANKRD52, HOXC10, KNOP1, and TRIM45 were significantly higher in MCF-10A cells than other cell lines, while SGPP1 was the opposite (Figure 10B). The results of Western blotting also verified the results of the database (Figure 10C).

## Discussion

Cuproptosis was recently defined as a distinct type of regulated cell death that occurs via a novel mechanism directly involving the TCA cycle of mitochondrial metabolism. Conversely, Ferroptosis, characterized by iron dependence, lipid peroxidation, and sensitivity to lipophilic antioxidants, is a well-researched regulated cell death. Literature suggests a relationship between Ferroptosis and TCA cycle with dysregulation of the TCA cycle leading to the occurrence of Ferroptosis. Additionally, TCA cycle metabolites serve as crucial sources of peroxide production, playing a role in the occurrence of Ferroptosis (Yang et al., 2022). Moreover, Gao et al. (Gao et al., 2019) demonstrated the reduction of Ferroptosis by inhibiting the mitochondrial TCA cycle.

According to the World Health Organization, in 2018 alone, 26,000 women worldwide were diagnosed with breast cancer. In the United States and China, the incidence and death rates of breast cancer vary among different ethnicities and races. White American women have an incidence rate of 133.7 per 100,000, while Chinese women have an incidence rate of 29.18 per 100,000. The death rates are 19.7 and 6.59 per 100,000 for White American and Chinese women, respectively (Giaquinto et al., 2022). Due to the high degree of heterogeneity of breast cancer based on genetic status and molecular subtypes, there are significant prognostic differences among patients with various subtypes. Therefore, the discovery of new prognostic biomarkers or models to guide clinical diagnosis and treatment is essential.

Currently, cuproptosis, a recently discovered cell death mechanism, has been studied in BRCA through the use of bioinformatics approaches (Li Z. et al., 2022; Sha et al., 2022; Zou et al., 2022). However, this study innovatively links cuproptosis and ferroptosis to construct a robust prognostic model for BRCA. In this study, we screened CFRGs obtained from GCTA and GEO databases using WGCNA and X-tile. Next, LASSO was implemented to select the optimal lambda value and five OCFRGs- ANKRD52, HOXC10, KNOP1, SGPP1, and TRIM45 were selected to build the model. These genes may play an important role in the regulation of cuproptosis and ferroptosis, or have a direct impact on the copper and iron metabolism of cells. ANKRD52 is reported to function as a tumor metastasis suppressor in lung adenocarcinoma. In addition to being a member of the PP6 holoenzyme, ANKRD52 inhibits tumor progression through PP6c-mediated dephosphorylation of PAK1 (Lee et al., 2021). ANKRD52 is reported to function as a tumor metastasis suppressor in lung adenocarcinoma. However, t Our study has identified a strong correlation between significantly elevated ANKRD52 expression levels in the BRCA and a poor prognosis. ANKRD52 showed a positive correlation with critical clinical indicators including ER, PR, and HER2, and had a high-risk score in our built model. Furthermore, we found that ANKRD52 expression was inversely correlated with the infiltration of Th1 cells, a subtype of antitumor immune cells.

Previous studies have confirmed that HOXC10 participates in the development and progression of breast cancer and affects the prognosis of breast cancer patients. The overexpression of HOXC10 in breast cancer is related to drug resistance to chemotherapy (Sadik et al., 2016) and a positive correlation between immune cell infiltration and poor prognosis in BRCAs (Zhang et al., 2022). Methylation of the HOXC10 promoter, resulting in transcriptional repression, has been shown to contribute to resistance to endocrine therapy in estrogen receptor-positive breast cancer (Pathiraja et al., 2014) In addition, studies have demonstrated that HOXC10 can promote tumor growth in BRCA by upregulating the level of IL-6 to activate the JAK2/STAT3 signal transduction pathway (Shen et al., 2022). HOXC10 expression is dysregulated in various cancers, acting as a carcinogenic driver associated with poor prognosis (Bao et al., 2014). In this study, highly expressed HOXC10 in breast cancer tissues and cells have been associated with high-risk scores, correlated with lymph node infiltration at the time of tumor detection, and positively associated with the infiltration of antitumor immune cells such as CD8^+^ T cells, NK cells, and DCs.

Studies have shown that the expression of SGPP1 is low in colorectal cancer and is correlated with tumor proliferation, migration, and invasion. Similarly, low expression of SGPP1 in triple-negative breast cancer strongly correlates with poor prognosis (Nema and Kumar, 2021). In addition, our study found that SGPP1 expression in breast cancer was associated with neutrophils, macrophages, CD4^+^ and CD8^+^ cells. Likewise, TRIM45 also acts as a tumor suppressor in brain tumors (Zhang et al., 2017) and non-small cell lung cancer (Peng et al., 2020). In this study, we found that the expression of TRIM45 in tumor tissues is higher than that in normal tissues, which correlated with a better prognosis. Furthermore, TRIM45 was highly positively correlated with clinical indicators ER and PR in breast cancer. However, research on KNOP1 in tumors is inadequate; therefore, more in-depth studies need to be conducted.

Single-cell RNA sequencing (scRNA-seq) analysis indicated that fibroblasts have high expression levels of HOXC10, KNOP1, and SGPP1. Cancer-associated fibroblasts (CAFs) are a significant constituent of the tumor microenvironment (TME) and have heterogeneous functions in cancer. Recent studies have demonstrated that targeted CAF therapy, combined with immunotherapy or chemotherapy, produces optimal therapeutic outcomes. For instance, the elevated expression of HOXC10, KNOP1, and SGPP1 in BRCA indicates that targeted CAF therapy, along with other treatments, might be more effective.

In this study, we developed a LASSO Cox model using the previously mentioned five OCFRGs, which successfully distinguished breast cancer patients into high-risk and low-risk groups. The model’s effectiveness was validated in three additional datasets, CGA Cohort, SE20685, and GSE58812, and its diagnostic efficiency was confirmed with a ROC curve analysis. Additionally, the model showed remarkable predictive utility for patient prognosis as a nomogram. Immune checkpoints and TMB are reliable immunotherapy markers. Our investigation of immune checkpoints and TMB in high- and low-risk groups revealed that the expression levels of immune checkpoints, including VTCN1, CD200R1, TNFRSF14, NRP1, TNFRSF4, CD40, CD200, CD44, TNFRSF25, CD48, and CD40LG, decreased in the BRCA high-risk group. Moreover, the risk score and TMB were positively correlated, with TIDE being lower in the high-risk group compared to the low-risk group. As such, the use of immune checkpoint inhibitors (ICIs) may enhance efficacy in high-risk BRCA. Previous research has demonstrated that ferritin therapy modifies tumor immunity (Wang et al., 2019), leading to immune cells within the tumor microenvironment (TME) to initiate lipid peroxidation and undergo ferroptosis, which can influence their function and survival. (Zuo et al., 2021). The use of ferroptosis inducers is a promising approach to augment the effectiveness of immune checkpoint inhibitor (ICI) therapy (Gao et al., 2022). In this study, the TIMER database was utilized to quantify immune cell infiltration in BRCA patients within the TCGA cohort. Our results indicated that Th1 and Th2 cells were higher within the high-risk group, whereas CD4^+^ T cells, CD8^+^ T cells, B cells, endothelial cells, and macrophages were lower.

Functional analysis revealed that genes within the high-risk group were primarily enriched in glycolysis, gluconeogenesis, the pentose phosphate pathway, and the cell cycle, whereas the MAPK signaling pathway was inhibited. Of these metabolic processes, glycolysis and gluconeogenesis are potential targets for cancer treatment. Additionally, the pentose phosphate pathway, which is a branch of glycolysis, plays a critical role in combating oxidative stress and assisting glycolytic cancer cells meet anabolic demands (Bao et al., 2014). The cell cycle is related to tumor cell proliferation, and heightened cell cycle activity in tumor cells inhibits antitumor activity. (Evan and Vousden, 2001). Moreover, the risk score was positively associated with mTOR, Hif-1, and ErbB pathways, among others, and negatively correlated with MAPK, PI3K/AKT, TGF-β, and Ras signaling pathways, among others. These results can aid in the investigation of the link between BRCA patients and signaling pathways within high- and low-risk groups. Some studies indicate that ferroptosis and cupperptosis may be targeted to treat tumors (Wang et al., 2021; Oliveri, 2022). Moreover, the risk score was positively associated with mTOR, Hif-1, and ErbB pathways, among others, and negatively correlated with MAPK, PI3K/AKT, TGF-β, and Ras signaling pathways, among others. These results can aid in the investigation of the link between BRCA patients and signaling pathways within high- and low-risk groups. Some studies indicate that ferroptosis and cupperptosis may be targeted to treat tumors.

In summary, we have systematically examined and discussed the molecular changes, intracellular pathways, and immune cell infiltration characteristics associated with cupperptosis and ferroptosis in breast cancer (BRCA) in our study. After utilizing bioinformatics techniques, we selected five OCFRGs (ANKRD52, HOXC10, KNOP1, SGPP1, and TRIM45), verified their differential expression in tumor and non-tumor biological tissue samples at both the cellular and tissue level, and then analyzed their relationship with tumor staging, cellular infiltration, and clinical indicators. The prognostic model we developed was able to reliably predict patient prognosis. Cell pathways closely related to ferroptosis and cupperptosis, such as glycolysis, gluconeogenesis, and the pentose phosphate pathway, were distinguished by the model as high-risk groups. The scoring model may offer guidance for clinical decision-making and precision treatment for BRCA patients.

## Conclusion

In conclusion, in this study we combined machine learning and bioinformatics methods to establish a prognostic model for 5 OCFRGs in BRCA. Based on this model, the prognosis in BRCA patients can be accurately predicted. In addition, the degree of immune infiltration and immune resistance in patients were also predicted. Our model guides clinicians in choosing the optimal treatment strategy to personalize treatment.

## Data Availability

Publicly available datasets were analyzed in this study. This data can be found here: TCGA-BRCA, GSE20685, GSE42568, GSE58812.
