# Supplementary material for: Cuproptosis/ferroptosis-related gene signature is correlated with immune infiltration and predict the prognosis for patients with breast cancer
Source: Front Pharmacol. 2023 Jul 13;14:1192434. doi: 10.3389/fphar.2023.1192434 (PMC10374203; doi:10.3389/fphar.2023.1192434)
Supplement: Supplementary file 1 [file DataSheet2.PDF]

gene	modt	lasso	morf	mogbdt	moxgboo[illegible]	imp_merge
REXO2	0.030689	0.057799	0.076104	0.105076	0.023361	0.058606
ADK	0.040271	0.013968	0.082329	0.113139	0.02192	0.054325
SNX3	0.03752	0.080544	0.075398	0.054314	0.020764	0.053708
ANKRD52	0.023394	0.094388	0.034033	0.065324	0.028233	0.049075
EMP1	0.036708	0.091119	0.048665	0.040231	0.025841	0.048513
MORF4L2	0.04595	0.04837	0.043378	0.060829	0.014723	0.04265
SGPP1	0.032616	0.069243	0.029253	0.025629	0.017682	0.034885
RCAN3	0.003246	0.048206	0.030012	0.050716	0.029732	0.032382
UBTF	0.011602	0.049818	0.036556	0.044205	0.014353	0.031307
DTX1	0.044052	0.052361	0.020391	0.015728	0.022102	0.030927
HSD11B1	0.024409	0.060805	0.020602	0.020183	0.019414	0.029083
SH2B2	0.02506	0.047554	0.006128	0.035623	0.024751	0.027823
HOXC10	0.032924	0.032481	0.025346	0.016598	0.022873	0.026044
CXCL2	0.016473	0.026791	0.014886	0.02584	0.046077	0.026013
TRIM45	0.025803	0.044831	0.020147	0.014167	0.019287	0.024847
IP6K2	0.010471	0.034227	0.031544	0.02392	0.021986	0.02443
SNRNP70	0.05241	0	0.022737	0.025626	0.018291	0.023813
CCNT1	0.024784	0.001966	0.026816	0.036002	0.022816	0.022477
ANXA5	0.025282	0.057006	0.007051	0.003939	0.016518	0.021959
RGL3	0.015997	0.042309	0.01222	0.009991	0.028933	0.02189
SUGP1	0.052814	0	0.014286	0.020777	0.019304	0.021436
SFI1	0	0	0.019316	0.063385	0.016469	0.019834
CCDC24	0.026044	0	0.036262	0.007271	0.022936	0.018503
KNOP1	0.032488	0.010333	0.018401	0.007943	0.022024	0.018238
THAP7	0.014035	0	0.018055	0.028849	0.027537	0.017695
EML1	0.023953	0.02537	0.009038	0.009106	0.019964	0.017486
OSTM1	0.013186	0	0.030644	0.012225	0.021976	0.015606
MYL5	0.021579	0	0.027201	0.011441	0.012691	0.014582
PIR	0.005993	0.01051	0.017427	0.00895	0.027652	0.014106
KDM4B	0.016012	0	0.021607	0.0026	0.028006	0.013645
