# Supplementary material for: Cuproptosis/ferroptosis-related gene signature is correlated with immune infiltration and predict the prognosis for patients with breast cancer
Source: Front Pharmacol. 2023 Jul 13;14:1192434. doi: 10.3389/fphar.2023.1192434 (PMC10374203; doi:10.3389/fphar.2023.1192434)
Supplement: Supplementary file 2 [file DataSheet4.PDF]

						Normalized
repeat1	GAPDH	190.637	175.76	153.876	197.111	
	HOXC10	109.508	186.664	160.339	183.836	0.574432
	TRIM45	63.165	261.3	192.405	133.947	0.576807
repeat2	GAPDH	118.383	105.412	90.624	120.863	
	HOXC10	82.283	139.832	123.523	148.088	0.695058
	TRIM45	54.324	161.91	150.086	122.55	0.660209
repeat3	GAPDH	180.032	164.298	143.856	187.678	
	HOXC10	104.476	154.874	150.76	175.485	0.580319
	TRIM45	58.234	154.291	144.007	110.743	0.557391

1.062039	1.042001	0.932652
1.399841	1.199989	0.728622

1.326528	1.363027	1.225255
1.157889	1.215045	0.827548

0.942641	1.047992	0.935032
0.996236	0.955207	0.631068
