# Supplementary material for: Cuproptosis/ferroptosis-related gene signature is correlated with immune infiltration and predict the prognosis for patients with breast cancer
Source: Front Pharmacol. 2023 Jul 13;14:1192434. doi: 10.3389/fphar.2023.1192434 (PMC10374203; doi:10.3389/fphar.2023.1192434)
Supplement: Supplementary file 3 [file DataSheet3.PDF]

KNOP1-F:CAGGAAGTGGACGGGAA
KNOP1-R:TGAACGAAGGGGACAGG
HOXC10-F:ACATCTGGAATCGCCTCA
HOXC10-R:CCCTCTCCTTTTCGCTCT
TRIM45-F:GTTGCCGTTGTCCCTAAA
TRIM45-R:GTTCTTTGATGCAGACCCA
SGPP1-F:GTTCCGTTATCATGTCGCT
SGPP1-R:CCTCCGCCTTTCTCATCC
ANKRD52-F:TGTCCCCATCCTCCAGTT
ANKRD52-R:CCCAGCACCTTCTCGTTT
GAPDH-F:CCTTCCGTGTCCCCACT
GAPDH-R:GCCTGCTTCACCACCTTC
