# Supplementary material for: Cuproptosis/ferroptosis-related gene signature is correlated with immune infiltration and predict the prognosis for patients with breast cancer
Source: Front Pharmacol. 2023 Jul 13;14:1192434. doi: 10.3389/fphar.2023.1192434 (PMC10374203; doi:10.3389/fphar.2023.1192434)
Supplement: Supplementary file 4 [file DataSheet1.PDF]

ID	TISSUE	ANKRD5	ANKRD5	HOXC10-	HOXC10-	KNOP1-st	KNOP1-p	TRIM45-s
A01	Tumor	3.2	2.4	2.8	3.2	3.8	3	2.8
A02	Normal	2	2	1.4	1.8	1.4	2	2
A03	Tumor	3	2.4	3	2.8	3.6	3.6	3.4
A04	Normal	2.2	1.8	2.4	2	1.6	2	1.4
A05	Tumor	4	3.4	2.8	3	3	2.8	3.2
A06	Normal	1	2	1.4	1	1.8	2	1.6
A07	Tumor	2.8	2.4	2.6	2.8	3.6	3.6	2.8
A08	Normal	2.2	2.2	2	1.2	1	1.4	1.6
A09	Tumor	3.6	3.4	2.8	3.2	3.8	2.8	2.8
A10	Normal	1.6	2.6	1.8	1.8	1.8	1.8	2
A11	Tumor	3.4	2.8	2.8	3.2	3.8	3.2	3.4
A12	Normal	1.2	2.4	2	1	1.2	1.6	1.6
B01	Tumor	3.2	2.6	3	3.2	3.6	3.6	3.2
B02	Normal	2.4	1.4	2.4	1.4	1.6	1.6	1.2
B03	Tumor	2.8	2.4	3.4	2.6	3.8	2.8	3.2
B04	Normal	1.8	2.6	1.8	1.4	1	1.6	1.2
B05	Tumor	3.6	2.2	2.8	3.2	3.4	3	3.4
B06	Normal	1	2.2	1.4	1	1.2	1.4	2
B07	Tumor	3.4	2.6	3	3.2	3.8	3.4	3
B08	Normal	1.2	1.2	1.4	2.2	1.2	1.6	2
B09	Tumor	3.4	2.4	3.2	2.6	3.6	2.8	3.4
B10	Normal	1.6	2.2	1.6	1.2	1	1.4	2
B11	Tumor	3.4	3.2	3.2	2.8	3.4	3.6	3.4
B12	Normal	1.6	2.6	1.4	1.4	1.6	1.6	1.2
C01	Tumor	3.4	3.2	2.8	3	3.4	3.2	2.8
C02	Normal	1.6	1.8	1.4	1	1.4	1.8	2
C03	Tumor	3.6	2.8	2.8	2.8	3	3	3.4
C04	Normal	1.8	2.2	2	1.4	1.8	1.6	1
C05	Tumor	2.8	2.6	3	2.8	3.8	3.4	3.4
C06	Normal	1.4	2.2	2	1	1.6	1.6	1.2
C07	Tumor	3.8	3	3.2	2.8	3.4	3.4	3.4
C08	Normal	2.2	1.2	2.4	2	1.2	2	1.2
C09	Tumor	3.8	3.2	2.8	2.6	3.2	3.6	2.8
C10	Normal	1.6	2.4	1.6	1.8	1.6	1.4	1.8
C11	Tumor	3.8	2.4	3.4	2.8	3	3.4	3.2
C12	Normal	1.6	2	2.2	1.6	1.2	1.6	1.2
D01	Tumor	3	2.6	3	3	3.6	3.2	3
D02	Normal	1	1.8	2.4	1	1	1.8	2
D03	Tumor	3.6	2.4	2.8	3.2	3.2	3.4	3
D04	Normal	1.8	2.4	2.4	1.8	1.6	1.4	1.8
D05	Tumor	3.4	3.2	2.8	3	3.2	3.4	3.2
D06	Normal	1.8	1.8	2.2	2.2	1	1.6	1.2
D07	Tumor	2.8	2.2	3.4	3	3.2	2.8	2.8
D08	Normal	2.2	2.6	2.2	2.2	1	1.8	1.6
D09	Tumor	3.8	2.4	3.4	3	3.4	3	3
D10	Normal	2.4	1.8	2.2	1.8	1.4	2	1.4
D11	Tumor	3.2	2.2	2.6	2.8	3.2	2.8	3.2
D12	Normal	1.8	1.2	1.4	1.4	1.6	1.6	1.4
E01	Tumor	2.8	3.2	2.6	3.2	3.6	2.8	3.4
E02	Normal	1	2.6	1.6	2	1.4	1.8	1.8
E03	Tumor	4	2.4	2.6	3.2	3	3.2	3.4
E04	Normal	1.2	2.6	1.6	2	1.8	1.8	1.2

E05	Tumor	3.8	2.6	3.4	2.8	3.8	3.4	3.2
E06	Normal	1.4	2	1.8	1.2	1.4	1.4	1.2
E07	Tumor	3	3	3	2.6	3	2.8	3.4
E08	Normal	2.2	2.4	2.2	1	1.2	1.8	2
E09	Tumor	2.8	3.2	3.4	3	3.6	3.2	3.2
E10	Normal	1.2	1.8	1.4	1.6	1.4	1.8	1.8
E11	Tumor	3.6	2.2	3	2.8	3.8	3.6	3
E12	Normal	2	2.4	1.8	2	1.4	1.4	1.2
F01	Tumor	4	2.6	3.4	2.6	3.6	3.6	3
F02	Normal	1.2	2.2	2.2	1.8	1	1.8	1.8
F03	Tumor	3.8	2.4	3	2.8	3	3.6	3
F04	Normal	2.4	2	2.2	2	1.8	1.4	1.8
F05	Tumor	3.8	2.6	2.6	2.8	3.2	3.6	3.4
F06	Normal	1.8	1.4	1.8	1.6	1.4	1.8	1.8
F07	Tumor	3.8	3.2	3.4	2.8	3.4	3	3.4
F08	Normal	2	2	1.8	2	1	1.4	1.6
F09	Tumor	4	3.2	2.6	3	3.6	3.2	3.2
F10	Normal	1.2	1.2	2.4	1	1.8	2	2
F11	Tumor	3.6	3.2	3.4	2.6	3.2	3	3
F12	Normal	1	1.8	2.4	2	1.4	2	1
G01	Tumor	2.8	2.8	2.8	3.2	3.2	3	3
G02	Normal	1.8	1.2	2.2	1.8	1	1.8	2
G03	Tumor	3.8	3.4	3.4	3.2	3.2	3.6	3.2
G04	Normal	1	2	1.4	2.2	1.2	1.6	2
G05	Tumor	4	3.4	3.4	3	3.6	3.6	3
G06	Normal	2.4	2.4	2.2	1	1.8	1.6	1.6
G07	Tumor	3.6	2.6	2.8	2.6	3.4	2.8	2.8
G08	Normal	2	1.4	2.4	2.2	1	1.6	1
G09	Tumor	2.8	3	3.4	2.8	3.4	3.6	3.2
G10	Normal	2.2	2	1.4	1.6	1.4	1.8	1.2
G11	Tumor	2.8	2.8	3.4	3.2	3.2	3	3.4
G12	Normal	1.8	1.2	2	2.2	1	1.6	1.6
H01	Tumor	3.4	2.6	3	3.2	3.4	3.6	2.8
H02	Normal	1.8	1.4	2.2	2	1.8	1.8	1.2
H03	Tumor	3.2	3.2	3	2.6	3.8	3.2	2.8
H04	Normal	2	2	2.2	2.2	1	1.4	1.4
H05	Tumor	3.2	3.4	2.6	3.2	3.8	3.4	3.2
H06	Normal	1.2	1.2	2	1.2	1.8	1.6	2

TRIM45-p	SGPP1-st	SGPP1-p	ANKRD5	HOXC10-	KNOP1-s	TRIM45-s	SGPP1-sum
3.4	2	2.2	5.6	6	6.8	6.2	4.2
1.6	3.8	3.2	4	3.2	3.4	3.6	7
3.2	2	2.2	5.4	5.8	7.2	6.6	4.2
1.4	3.2	3	4	4.4	3.6	2.8	6.2
2.8	1.8	1.8	7.4	5.8	5.8	6	3.6
1	3.6	3.4	3	2.4	3.8	2.6	7
3	1.6	1.6	5.2	5.4	7.2	5.8	3.2
1	3.8	3	4.4	3.2	2.4	2.6	6.8
3	1.4	1.8	7	6	6.6	5.8	3.2
1.8	3	3.2	4.2	3.6	3.6	3.8	6.2
3.2	1.6	1.4	6.2	6	7	6.6	3
1.4	3.2	2.8	3.6	3	2.8	3	6
2.8	2	1.4	5.8	6.2	7.2	6	3.4
1.6	3.6	3.6	3.8	3.8	3.2	2.8	7.2
3	2.4	1.2	5.2	6	6.6	6.2	3.6
1.2	3.6	3.2	4.4	3.2	2.6	2.4	6.8
3	2	2	5.8	6	6.4	6.4	4
1.2	3.2	3.6	3.2	2.4	2.6	3.2	6.8
2.6	1.8	1.8	6	6.2	7.2	5.6	3.6
1.8	3.4	3.6	2.4	3.6	2.8	3.8	7
3.4	1.6	1.8	5.8	5.8	6.4	6.8	3.4
2	3.4	3.4	3.8	2.8	2.4	4	6.8
3.4	1.4	1.8	6.6	6	7	6.8	3.2
1.6	3.4	3	4.2	2.8	3.2	2.8	6.4
3	1.8	1.6	6.6	5.8	6.6	5.8	3.4
1.4	3.8	3.4	3.4	2.4	3.2	3.4	7.2
3.6	2	1.4	6.4	5.6	6	7	3.4
1.8	3.8	3.4	4	3.4	3.4	2.8	7.2
3.2	2.2	1.4	5.4	5.8	7.2	6.6	3.6
2.2	4	3.2	3.6	3	3.2	3.4	7.2
2.6	2	1.4	6.8	6	6.8	6	3.4
1.2	4	3.6	3.4	4.4	3.2	2.4	7.6
2.8	2.4	2.2	7	5.4	6.8	5.6	4.6
1.4	3.8	3.2	4	3.4	3	3.2	7
3.4	1.8	1.4	6.2	6.2	6.4	6.6	3.2
2	3	3	3.6	3.8	2.8	3.2	6
3.6	1.4	1.6	5.6	6	6.8	6.6	3
1.6	4	2.8	2.8	3.4	2.8	3.6	6.8
3.6	2.2	1.4	6	6	6.6	6.6	3.6
1	3.2	3.2	4.2	4.2	3	2.8	6.4
3.2	1.8	1.2	6.6	5.8	6.6	6.4	3
2.2	3.6	3.4	3.6	4.4	2.6	3.4	7
3.4	2.2	1.2	5	6.4	6	6.2	3.4
2.2	3	3.2	4.8	4.4	2.8	3.8	6.2
3.6	1.8	1.4	6.2	6.4	6.4	6.6	3.2
1.8	4	3.6	4.2	4	3.4	3.2	7.6
2.8	1.6	1.4	5.4	5.4	6	6	3
1	4	2.8	3	2.8	3.2	2.4	6.8
3	1.8	1.8	6	5.8	6.4	6.4	3.6
2	3.6	3	3.6	3.6	3.2	3.8	6.6
2.6	2	2.4	6.4	5.8	6.2	6	4.4
1.2	3.2	2.8	3.8	3.6	3.6	2.4	6

3.6	1.6	1.6	6.4	6.2	7.2	6.8	3.2
1.8	3.6	2.8	3.4	3	2.8	3	6.4
3	2	1.2	6	5.6	5.8	6.4	3.2
1	4	3.2	4.6	3.2	3	3	7.2
3.6	1.6	2	6	6.4	6.8	6.8	3.6
1	3.8	3	3	3	3.2	2.8	6.8
3.4	1.4	1.8	5.8	5.8	7.4	6.4	3.2
1.8	3	2.8	4.4	3.8	2.8	3	5.8
3.4	2.2	2.4	6.6	6	7.2	6.4	4.6
1.4	3.8	3.2	3.4	4	2.8	3.2	7
2.6	2	1.4	6.2	5.8	6.6	5.6	3.4
2.2	4	2.8	4.4	4.2	3.2	4	6.8
2.6	2	1.6	6.4	5.4	6.8	6	3.6
1.4	3.4	2.8	3.2	3.4	3.2	3.2	6.2
2.6	2.2	2.4	7	6.2	6.4	6	4.6
2.2	4	3	4	3.8	2.4	3.8	7
2.6	1.8	2	7.2	5.6	6.8	5.8	3.8
1.6	3.6	3.4	2.4	3.4	3.8	3.6	7
2.6	1.6	1.2	6.8	6	6.2	5.6	2.8
1	3.2	3.2	2.8	4.4	3.4	2	6.4
2.8	2.2	2.4	5.6	6	6.2	5.8	4.6
1	3.6	3.6	3	4	2.8	3	7.2
2.6	1.4	1.2	7.2	6.6	6.8	5.8	2.6
1.6	3.2	3.2	3	3.6	2.8	3.6	6.4
3.2	1.6	1.4	7.4	6.4	7.2	6.2	3
1.2	3.8	3.4	4.8	3.2	3.4	2.8	7.2
3.6	2.4	1.4	6.2	5.4	6.2	6.4	3.8
1.6	3.6	3.4	3.4	4.6	2.6	2.6	7
3	2.4	1.6	5.8	6.2	7	6.2	4
2.2	4	3.2	4.2	3	3.2	3.4	7.2
3.2	2.4	2.4	5.6	6.6	6.2	6.6	4.8
1.4	3.2	3.4	3	4.2	2.6	3	6.6
2.6	1.4	1.4	6	6.2	7	5.4	2.8
1.6	3.8	3.4	3.2	4.2	3.6	2.8	7.2
2.8	2.2	1.8	6.4	5.6	7	5.6	4
1	3.8	3.6	4	4.4	2.4	2.4	7.4
3.6	2.2	1.8	6.6	5.8	7.2	6.8	4
1.8	3.6	3	2.4	3.2	3.4	3.8	6.6
